# Cine DENSE MRI for circumferential and radial dyssynchrony in patients referred for cardiac resynchronization therapy

**DOI:** 10.1186/1532-429X-11-S1-O90

**Published:** 2009-01-28

**Authors:** Alexander B Jehle, Frederick H Epstein, Xiaodong Zhong, Robert L Janiczek, W Kevin Tsai, John M Christopher, Dale E Fowler, John D Ferguson, Christopher M Kramer, Kenneth C Bilchick

**Affiliations:** University of Virginia, Charlottesville, VA USA

**Keywords:** Cardiac Resynchronization Therapy, Circumferential Strain, Cardiac Resynchronization Therapy Patient, Radial Contraction, Cardiac Resynchronization Therapy Candidate

## Introduction

Cine DENSE (Displacement Encoding with Stimulated Echoes) MRI offers high resolution circumferential, radial, and longitudinal strain assessment. Dyssynchrony assessment using midwall circumferential strain corresponds to the predominant physiologic orientation of myofibers, and its effectiveness for cardiac resynchronization therapy (CRT) patient selection has recently been demonstrated [1]. Current echocardiographic dyssynchrony measures are based on radial or longitudinal velocity/strain.

## Purpose

To test the hypothesis that cine DENSE circumferential strain analysis most effectively distinguishes between HF patients referred for CRT (HF/CRT) versus HF patients not candidates for CRT (HF/no CRT) and normal volunteers.

## Methods

MRI and echocardiography were performed in 21 subjects separated into 3 groups as defined above: HF/CRT patients (N = 9), HF/no CRT patients (N = 8), and normal volunteers (N = 4). All MRI studies were performed on a 1.5 T system (Avanto, Siemens, Germany). An ECG-gated spiral cine DENSE pulse sequence was used to acquire images with displacement encoding applied in two orthogonal in-plane directions. Separate 14-heartbeat breath-hold acquisitions were used for each displacement encoding direction. Short-axis images were acquired at basal, mid-ventricular, and apical levels. Additional imaging parameters included field of view = 340 – 400 mm^2^, matrix = 128 × 128, slice thickness = 8 mm, flip angle = 20°, TR = 17 ms, TE = 1 ms, number of spiral interleaves = 6, fat suppression, temporal resolution = 17 ms, and displacement encoding frequency = 0.1 cycles/mm. Images were exported to a PC and analyzed using custom-developed segmentation, tissue tracking, and strain analysis methods that have been described previously [2]. Echocardiography studies were performed on GE Vivid 7 Scanners and analyzed on an EchoPAC workstation. The standard deviations in timing in 12 segments were calculated for MR-based onset of circumferential contraction (MR Ecc Onset SD), peak circumferential contraction (MR Ecc Peak SD), onset of radial contraction (MR Err Onset SD), and peak radial contraction (MR Err Peak SD), as well as for echo peak longitudinal velocity (Ts-SD), as described by Yu et al [3].

## Results

**Figure 1:**
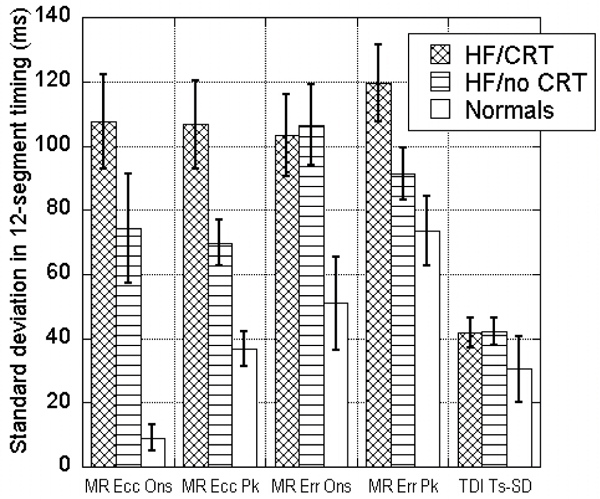
**CMR DENSE dyssynchrony measures**.

Of the 21 patients (age 60 +/- 11 years, 29% female), 17 had a cardiomyopathy, 6 (35%) of which had an ischemic etiology. For all 252 segments, DENSE circumferential and radial peak timing correlated modestly (R = 0.43; p < 0.0001). Patient-level circumferential timing measures for onset of circumferential contraction and peak circumferential contraction correlated closely (R = 0.79; p < 0.0001). As shown in Figure 1, MR Ecc Peak SD was significantly higher in HF/CRT patients versus HF/no CRT patients (p = 0.01) and normal patients (p = 0.008). MR Ecc Onset SD was markedly higher in HF/CRT patients than normals (p = 0.001). Although DENSE radial dyssynchrony measures effectively differentiated HF/CRT patients from normals, they were not as effective as the circumferential measures in distinguishing between patients in the HF/CRT and HF/no CRT groups (p = NS). Ts-SD as assessed by echocardiography was similar in all 3 groups.

## Conclusion

MR cine DENSE assessment of circumferential strain timing using contraction onset and peak contraction effectively distinguished CRT candidates from HF patients not candidates for CRT and normal volunteers. The better discrimination of circumferential versus radial strain dyssynchrony measures has important implications for identifying the optimal imaging modality to identify CRT candidates.
